# Do conservation strategies that increase tiger populations have consequences for other wild carnivores like leopards?

**DOI:** 10.1038/s41598-019-51213-w

**Published:** 2019-10-11

**Authors:** Ujjwal Kumar, Neha Awasthi, Qamar Qureshi, Yadvendradev Jhala

**Affiliations:** Wildlife Institute of India, Chandrabani, Dehradun, 248001 India

**Keywords:** Conservation biology, Conservation biology, Conservation biology, Conservation biology, Population dynamics

## Abstract

Most large carnivore populations are declining across their global range except in some well managed protected areas (PA’s). Investments for conserving charismatic apex carnivores are often justified due to their umbrella effect on biodiversity. We evaluate population trends of two large sympatric carnivores, the tiger and leopard through spatially-explicit-capture-recapture models from camera trap data in Kanha PA, India, from 2011 to 2016. Our results show that the overall density (100 km^−2^) of tigers ranged between 4.82 ± 0.33 to 5.21 ± 0.55SE and of leopards between 6.63 ± 0.71 to 8.64 ± 0.75SE, with no detectable trends at the PA scale. When evaluated at the catchment scale, Banjar catchment that had higher prey density and higher conservation investments, recorded significant growth of both carnivores. While Halon catchment, that had lower prey and conservation investments, populations of both carnivores remained stable. Sex ratio of both carnivores was female biased. As is typical with large carnivores, movement parameter sigma (an index for range size), was larger for males than for females. However, sigma was surprisingly similar for the same genders in both carnivores. At home-range scale, leopards achieved high densities and positive growth rates in areas that had low, medium or declining tiger density. Our results suggest that umbrella-species conservation value of tigers is likely to be compromised at very high densities and therefore should not be artificially inflated through targeted management.

## Introduction

The tiger, once widely distributed across Asia has now lost 93% of its former range^1^ and currently occurs only in 11 countries^2^. India holds the largest wild tiger population estimated at about of 70% of the global population^3^. Due to its charisma, the tiger attracts conservation investments from Governments and civil society. Being an apex predator, it also serves as an umbrella for conserving Asia’s forest biodiversity^4^. The leopard is more widely distributed across much of Africa and parts of Asia. Its status is less precarious compared to tigers, with whom the leopards are sympatric in Asia. However, despite being a generalist, capable of exploiting a multitude of habitats, prey, and adapted to live in close proximity with people^5^ the leopard is still on the decline globally^6^.

India has made large investment in the form of Protected Areas, human resettlement, law enforcement and habitat management to conserve tigers so as to reap their benevolent umbrella effect in protecting biodiversity^7^ and ecosystem services^8^. Tigers being apex predators out-compete and often kill other predators like leopards, dhole, and sloth bear^9^. In areas of high tiger densities, leopard are likely out-competed^10^. High tiger density areas like Corbett and Kaziranga National Parks though having abundant prey, have very few leopards^3,11^. Habitats without refuge from tigers are devoid of leopards e.g. the mangrove swamp forests of Sundarbans. Understanding population response of leopards at various ecologically relevant spatial scales to tigers would provide insights on thresholds of the benevolent effect of tigers on sympatric large carnivores and at what densities tigers become detrimental to other threatened carnivores.

Often abundance estimates and population trends of threatened species are required for evaluating the success of management actions and prioritising conservation investments^12^. Despite their ecological importance, there are limited studies on long-term population trends of large carnivores^13^. Development of camera trap based classical capture-recapture^14,15^ is the method of choice for estimating abundance of uniquely identifiable individuals of a species e.g. tiger^15^, jaguar^16^, leopard^10^ and ocelots^17^. Most studies on tigers and leopards are limited to assessment of their abundance^3,10,15,18^, while studies that address other demographic parameters are rare^19–22^. Most population trend studies on tigers use either classical non-spatial closed capture-recapture models^21,23^ or statistically less rigorous track counts^24^. Herein, we use spatially explicit capture recapture (SECR)^25,26^ to estimate tiger and leopard spatial densities, sex ratios^27^ and their trends over a six-year period in Kanha National Park, a major stronghold for both species in Central India. We demonstrate the importance of monitoring, both at local and PA scales, so as to gain an understanding of spatial population dynamics and guide conservation management with site specific information. We analyse our data at home-range scales to gain insights into how leopards and tigers interact over time and space.

## Results

With an annual effort ranging between 3992 to 34868 trap nights we photo-captured 125 adult tigers that included 61 males and 64 females; 217 adult leopards that included 78 males, 120 females and 19 of unknown gender. Total annual photo-captures for tigers for the PA were between, 122 to 1584 and for leopard between, 57 to 935. The mean number of captures for an individual tiger were 9 (range 1 to 45) photo-captures, while for leopards were 3.8 (range 1 to 20) photo-captures. None of the individuals in the study were photo-captured in Banjar as well as Halon catchments within the same year, suggesting a clear separation between sites in the short-term. However, 04 tigers and 01 leopards were observed to have dispersed between catchments between sampling periods (years). The mean maximum (±SE) distance moved by tigers was 6.5 ± 0.48 km (Max 25 km), for leopards the estimate was 5.16 ± 0.60 km (Max 28.6 km; Table S1). The gender of all tigers and 91% of leopards were identified from camera trap photos.

The best model for both tigers and leopards for the entire data representing Kanha National Park as well as for individual catchments was the same, wherein variation in g_0_ and σ were explained by sex as well as sampling years (Table S2 & S3). Tiger density (±SE) of Kanha PA (at 100 km^−2^) ranged between 4.82 ± 0.33 to 5.21 ± 0.55 and leopard densities (at 100 km^−2^) between 6.63 ± 0.71 to 8.64 ± 0.75 (Table 1). Since variation in sex ratio between years did not contribute to explaining our data and was therefore not selected in the top model, we report the overall sex ratio for each catchment (except for leopard estimates in Banjar catchment, Table 1). Overall sex ratios in Kanha National Park (M:F) was biased towards females for both tigers (0.66 ± 0.03) and leopards (0.50 ± 0.02).

**Table 1:** Density (±SE at 100 km^−2^), detection probability (g_0_), spatial scale of detection (σ km), and detection corrected sex ratio of tigers and leopards in Kanha National Park, Halon and Banjar Catchments.

Site	Sampling Year	Tiger						Leopard					
		Density (100 km^−2^)	g_0_ Female	g_0_ Male	σ (km) Female	σ (km) Male	Sex Ratio (M: F)	Density (100 km^−2^)	g_0_ Female	g_0_ Male	σ (km) Female	σ (km) Male	Sex Ratio (M: F)
Banjar Catchment	2011	6.34 ± 0.80	0.07 ± 0.003	0.05 ± 0.001	1.59 ± 0.05	2.56 ± 0.08	0.55 ± 0.03	4.36 ± 0.76	0.003 ± 0.001	0.003 ± 0.001	1.39 ± 0.09	2.98 ± 0.2	0.45 ± 0.09
	2012	6.99 ± 0.64			1.86 ± 0.07	3.00 ± 0.10		5.30 ± 0.66			1.64 ± 0.12	2.71 ± 0.08	0.37 ± 0.08
	2013	7.70 ± 0.53			1.64 ± 0.06	2.64 ± 0.09		6.10 ± 0.57			1.30 ± 0.08	2.58 ± 0.08	0.35 ± 0.08
	2014	8.49 ± 0.54			1.41 ± 0.02	2.27 ± 0.05		7.03 ± 0.59			0.79 ± 0.04	1.66 ± 0.07	0.54 ± 0.08
	2015	9.36 ± 0.74			1.39 ± 0.03	2.25 ± 0.05		8.09 ± 0.80			0.78 ± 0.05	1.68 ± 0.09	1.86 ± 0.09
	2016	10.32 ± 1.09			1.27 ± 0.03	2.04 ± 0.04		9.30 ± 1.26			1.04 ± 0.04	2.23 ± 0.08	0.54 ± 0.07
Halon Catchment	2011	2.27 ± 0.50	0.05 ± 0.003	0.04 ± 0.003	2.64 ± 0.16	3.74 ± 0.28	0.66 ± 0.06	4.98 ± 0.97	0.04 ± 0.004	0.03 ± 0.002	1.21 ± 0.11	2.48 ± 0.20	0.46 ± 0.04
	2012	2.24 ± 0.37			2.30 ± 0.12	3.25 ± 0.23		5.32 ± 0.80			1.23 ± 0.12	2.54 ± 0.21	
	2013	2.21 ± 0.29			2.46 ± 0.15	3.58 ± 0.23		6.00 ± 0.67			1.17 ± 0.09	2.41 ± 0.20	
	2014	2.19 ± 0.29			2.21 ± 0.89	3.13 ± 0.14		6.59 ± 0.64			1.05 ± 0.08	2.16 ± 0.20	
	2015	2.16 ± 0.36			2.40 ± 0.16	3.39 ± 0.19		7.24 ± 0.82			1.01 ± 0.06	2.08 ± 0.13	
	2016	2.13 ± 0.47			2.08 ± 0.87	2.95 ± 0.13		7.95 ± 1.19			1.34 ± 0.07	2.76 ± 0.13	
Kanha National Park	2013	5.21 ± 0.55	0.04 ± 0.001	0.03 ± 0.0008	2.60 ± 0.50	3.03 ± 0.75	0.66 ± 0.03	6.63 ± 0.71	0.018 ± 0.001	0.02 ± 0.001	1.40 ± 0.06	2.52 ± 0.09	0.50 ± 0.02
	2014	5.01 ± 0.34			1.59 ± 0.23	2.37 ± 0.45		7.24 ± 0.49			1.34 ± 0.04	2.44 ± 0.06	
	2015	4.87 ± 0.33			1.95 ± 0.35	2.91 ± 0.52		7.90 ± 0.46			1.59 ± 0.63	2.86 ± 0.08	
	2016	4.82 ± 0.33			1.85 ± 0.30	2.76 ± 0.44		8.64 ± 0.75			1.84 ± 0.50	2.92 ± 0.07	

Mean abundance of tigers at the PA scale showed an annual decline of 2% (R^2^ = 0.94; P = 0.002) and leopards showed an annual increase of 8.8% (R^2^ = 0.99, P < 0.001) between 2013 to 2016. However, after considering the variability in the abundance estimates, the 95% confidence intervals on λ showed no detectible trends for both carnivores (95% CI on tiger λ = 0.86 to 1.07 and (95% CI on leopards λ = 0.99 to 1.20). Density for both tigers and leopards in Banjar catchment showed growth with a λ = 1.10 (*CI*_*95%*_ 1.02–1.18; R^2^ = 0.99, P < 0.001) for tigers and λ = 1.15, (*CI*_*95%*_ 1.05–1.27; R^2^ = 0.99, P < 0.001) for leopards. For the Halon catchment tiger density remained stable at λ = 0.98 (*CI*_*95%*_ 0.86–1.14) while leopard density increased at λ = 1.09 but was statistically insignificant (*CI*_*95%*_ 0.98–1.22).

**Figure 1:**
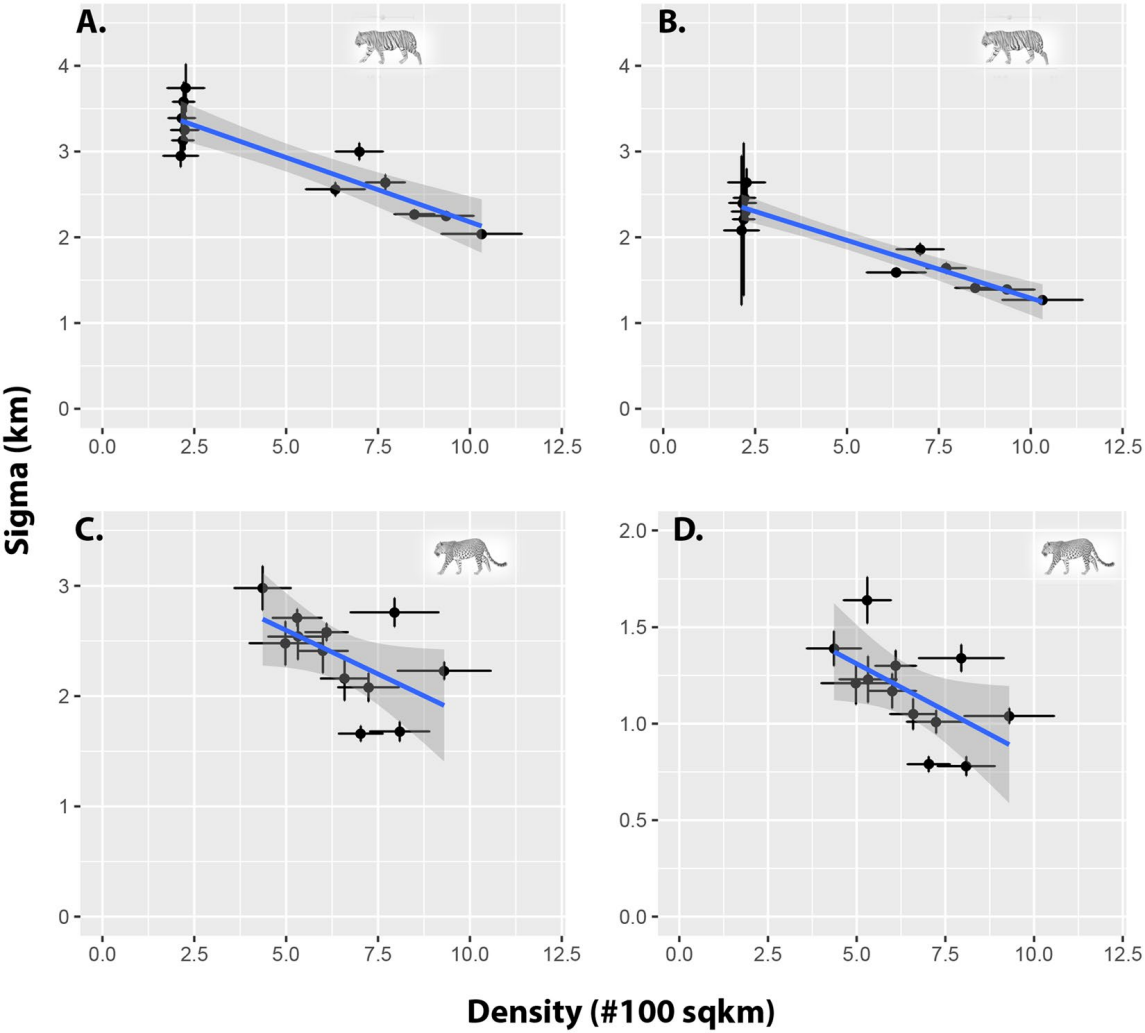
Relationship between Sigma σ (km ± SE), an index of home-range *vs* Density (at 100 km^−2^ ±SE), (**A**) female tigers, (**B**) male tigers, (**C**) female leopards and (**D**) male leopards.

Sigma, which is an index of home-range size was higher in males compared to females in both tigers and leopards (Table 1). Interestingly σ’s of tigers and leopards were similar for the same sex (Table 1). Average σ, the scale parameter estimated by SECR, showed a declining trend with increasing density for both genders of tigers (♂, *r* = −*0.93 &* ♀, *r* = −0.94; P < 0.001) and leopards (♂ = *r* − 0.56, P = 0.056; ♀ = *r* − 0.57, P = 0.05) (Fig. 1). Simulation results of 100 regression analysis showed that in all cases (both genders) for tigers and leopards the slope of the regression was negative (Table S4). The 95% confidence interval for the slope of the regression for both genders of tigers and leopards was negative and did not include zero. Therefore, we concede that the observed declining trend in σ with increasing density was genuine and not an artefact of sample variability.

**Figure 2:**
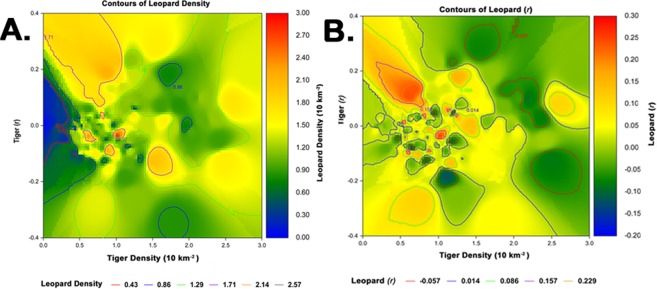
(**A**) Heat and contour map of leopard density plotted against tiger density and tiger growth rate (r). (**B**) Heat and contour map of leopard growth rate (r) plotted against tiger density and tiger growth rate. Density of both tiger and leopard was estimated by SECR and growth rate estimated for each 10 km^2^ pixel by regressing Ln(Density) against years.

At tiger home range scale (10 km^2^ grids), leopards achieved high density in areas with low, medium or declining tiger density. Leopard populations showed positive growth in areas with low to medium-stable or declining tiger density (Fig. 2).

## Discussion

Often placement of cameras is such that they attempt to maximize photo-captures of the target species, in our case the tiger. However, such placement that results in enhanced precision for tigers (more frequent recaptures) does not enhance photo-captures of leopards that often avoid the most frequented paths used by tigers^28^. The use of one sampling design, which can accommodate precise density estimates for both tigers and leopard, was initially a challenge. This was addressed by increasing camera density and placement of some cameras away from large trails and dirt tracks that tigers often use and leopards avoid. The SECR based density estimates are mostly robust to bias but require a proper sampling design for achieving good precision^29,30^. Based on the recommended sampling design^29,30^ for an unbiased density estimate, placement of camera traps should be at distances less than the home-range radius of the target species, while the size of the sampled area should be greater than 1.5 times the home-range size of the target species. Our study met these minimal criteria for both, tigers and leopards. The high density and small inter-camera distances ensured that each tiger/leopard had the potential to be exposed from one to several cameras and there were no “holes” in the study design.

The overall density of tigers and leopards in Kanha National Park did not show any trend. Density of leopards was significantly higher than tigers. The two sampling blocks, Banjar and Halon catchments, were selected based on substantial differences in prey densities and conservation investments with the objective of studying the response of large carnivores to these differences. Contrary to our expectation, both predators occurred at reasonably high densities and both showed positive growth in the Banjar catchment. Here, tigers density (at 100 km^−2^) significantly increased from 6.34 to 10.32, while leopard density (at 100 km^−2^) increased from 4.36 to 9.30 (Table 1). While in Halon catchment that had lower prey density and less investment in conservation management, both carnivores did not show a detectable increase. Tigers are more K-selected when compared to leopards^31^ and should exhibit slower life history traits manifesting in slower population growth when compared to leopards. But contrary to our expectations, the growth rate of leopards was comparable to that observed for tigers. This result is suggestive of competitive inhibition of leopards by the larger tiger. Besides competing for food (which was plentiful), tigers are known to pursue and kill leopards^9^. Leopard densities were significantly higher compared to tiger densities in Halon catchment and both carnivore populations did not show growth here. We believe that this was a due to high human disturbance and possibly due to poaching of prey and carnivores. Leopards fare better than the tiger when faced with poaching, since there is higher illegal demand for tiger body parts compared to leopards, and leopards have faster life history traits compared to tigers^31^. This was reflected in Halon catchment that had more leopards compared to tigers.

Home-range scales to body size^32^. Smaller carnivores are expected to have smaller ranges compared to larger carnivores especially when both have similar food habits and foraging strategy^33^. We therefore, expected leopards to have smaller σ compared to tigers. Our study showed contrary results where σ was similar for tigers and leopards. This suggests that leopards in Kanha Tiger Reserve had to invest almost as much in movement as tigers, that are three times larger in size. This further points to the high adaptability of leopards that not only survive well in human dominated landscapes^5^ but also do well in areas with high tiger density through higher investments (e.g. maintaining large home-ranges).

Efford *et al*.^34^ showed an exponential decline in σ with increasing tiger density at landscape scales^34^. Density dependent home range sizes have been published for many species^22,35,36^. These suggest that home-range size adjusts like an elastic disk to changes in density^37^ and when nearing carrying capacity the rate of decrease in home-range size is no longer possible (reaches an asymptote). Our local scale data for σ of both tigers and leopards showed a linear decline with density suggesting that the population of both tigers and leopards was not yet at carrying capacity and could potentially increase further.

In SECR the density surface depicted by the model (without covariates) is of the realized capture process and not the actual density estimated after correcting for capture probability^38^. In our case, since we had a high density of camera traps and a reasonable number of trap nights the difference between the photo-captured individuals (M_t+1_) and the population estimate $\hat{{\rm{N}}}$ were close (see Table 2) and therefore the surface depicting tiger and leopard photo-captures would be very close to the actual density. At the fine scale of home-range, our heat and contour plots clearly show that though leopards coexisted with tigers, they paid a price for this coexistence and achieved high density only in areas that had either low tiger density or where tiger density showed declines. Growing leopard populations were found in low tiger density areas or areas with declining tiger density (Fig. 2). All of the above independent population response assessments (large home-ranges, regions with high leopard density and growth at fine home range scale) point to the costs to leopards for being sympatric with tigers.

**Table 2:** Sampling effort, detections and population estimates of tigers and leopards within Kanha National Park, Banjar and Halon catchments between 2011 to 2016.

Site	Sampling Year	Camera Locations	Effort(Trap nights)	Average Trap distance (Km)	Tiger			Leopard		
					No. detections	Unique Tigers (M, F)	Population Estimate Realised ($\hat{N}$)	No. detections	Unique Leopards (M, F, UN)	Population Estimate Realised ($\hat{N}$)
Banjar Catchment	2011	58	2842	1.8	314	33 (13, 20)	35 ± 1.5	84	20 (9, 10, 1)	22 ± 1.66
	2012	58	2092	1.8	253	33 (13, 20)	34 ± 1.3	86	21 (9, 11, 1)	24 ± 2.0
	2013	58	2610	1.8	291	41 (16, 25)	44 ± 1.71	100	24 (9, 12, 3)	30 ± 2.76
	2014	279	8129	0.5	965	53 (23, 30)	54 ± 0.98	129	29 (13, 16, 0)	36 ± 2.70
	2015	140	4848	1.0	686	45 (19, 28)	48 ± 1.73	75	30 (23, 7, 0)	39 ± 3.34
	2016	140	6468	1.0	849	47 (23, 24)	51 ± 2.01	257	37 (17, 18, 2)	42 ± 2.43
Halon Catchment	2011	38	1368	1.8	122	10 (2, 8)	10 ± 0.19	61	15 (8, 7, 0)	17 ± 1.72
	2012	38	1900	1.8	192	12 (5, 7)	12 ± 0.17	57	14 (8, 6, 0)	16 ± 1.69
	2013	38	1824	1.8	121	10 (5, 5)	10 ± 0.13	72	16 (7, 8, 1)	19 ± 1.87
	2014	191	3831	0.5	276	14 (8, 6)	14 ± 0.08	91	19 (8, 11, 0)	21 ± 1.53
	2015	98	2346	1.0	165	12 (8, 4)	12 ± 0.09	115	25 (10, 14, 1)	27 ± 1.53
	2016	98	4280	1.0	206	9 (4, 5)	09 ± 0.08	269	28 (11, 17)	29 ± 0.85
Kanha National Park	2013	155	7595	1.8	542	60 (26, 34)	61 ± 0.9	250	57 (22, 28, 7)	71 ± 4.35
	2014	758	34868	1.8	1584	74 (37, 37)	74 ± 0.48	520	84 (35, 48, 1)	91 ± 2.71
	2015	384	15360	1.8	1179	62 (27, 35)	62 ± 0.49	335	85 (41, 40, 4)	91 ± 2.49
	2016	384	18816	0.5	1477	62 (27, 35)	62 ± 0.46	935	105 (38, 62, 5)	109 ± 2.17

M- male, F- female, UN- unidentified sex.

Earlier estimates using non-spatial CMR as well as most SECR estimates rarely take into account local variations in density, and population well-being is usually inferred from the overall response in density and its trends for a PA or catchment. This could mislead policy and management decisions^39^. Our study highlights the importance of estimating densities at various scales since diverse responses to differential management, prey populations and demography of apex carnivores are possible within the same PA. The low-density area of Halon catchment would benefit substantially by an increased protection regime and reduction of human disturbance. SECR when employed with a proper study design provides results required for site specific management of endangered species populations.

Often charismatic large carnivore populations, like those of Asiatic lions (*Panthera leo*) and tigers are intensively managed through habitat manipulations, health care interventions, baiting, and regulating social mortality in PA’s of India^22,40^. Such well-meaning management interventions can inflate local or PA densities of these apex carnivores that can often be detrimental to other carnivores, prey, and even alter the natural selection processes operating within these apex carnivores^40^. Our data shows that leopards can coexist with tigers, but with potentially high costs, we believe that similar response would be seen for sloth bear and dhole. The benevolent role of tiger conservation as an umbrella species would be best achieved by allowing natural process to which these species have adapted for sympatric coexistence, management should attempt to reduce human impacts within PA’s and refrain from the urge to increase the population of the apex carnivore by population and habitat manipulation beyond a point. In this study, using camera trap SECR with sex based heterogeneity models^27,41^ we have gained significant ecological insights on the likely role interactions, prey, and good protection play on population response of two sympatric large carnivores. Leopards response to tigers varied with scale, at PA and catchment scale, prey and protection regime were dominant factors determining the response of both carnivores. While at home-range scale, tiger density and demography depressed leopard demography.

## Methods

### Study area

Kanha National Park is one from the first group of seven tiger reserves established in 1973. It is situated in Mandla and Balaghat districts of Madhya Pradesh state in India. The Kanha National Park is 940 km^2^ and encompasses the catchments of two rivers, the Banjar and Halon. A narrow ridge of Bhaisanghat separates these two catchments. Since its inception as a National Park in 1955, the Banjar catchment gained considerable conservation investment where majority of the villages were relocated prior to 1998. Additionally control of poaching, law enforcement and habitat restoration in the form of water and grassland management by woody and invasive species removal is being practiced here. In the Halon catchment, which was added to the National Park in 1976, there was far less investment and human habitation resettlement was done more recently (last village resettled in 2017).

We believe, that as a result of higher interventions in the Banjar catchment, it has higher prey densities and biomass (33,963 kg/km^2^) and less anthropogenic pressure due to the absence of villages. While the Halon catchment has an ungulate biomass of 7257 kg/km^2^ ^42,43^. By 2017, most of the villages from Halon catchment were also relocated and conservationists are hopeful of prey recovery. The ungulate density of Kanha National Park was 50 ± 4.80/km^2^, which is one of the highest prey densities in Asian PA’s with a biomass of 26,806 ± 2,573 kg/km^2 44^.

### Methodology

**Figure 3:**
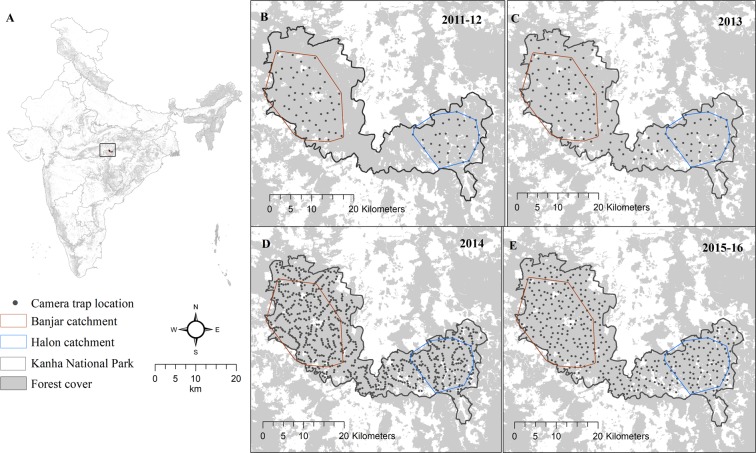
Study area and study design at Kanha National Park (**A**) Map showing location of Kanha National Park in India, Sampling design (**B**) in 2011 & 2012, (**C**) in 2013, (**D**) in 2014, and (**E**) in 2015 & 2016. Data from areas that were consistently sampled across all years (Banjar catchment -brown polygon, Halon catchment -blue polygon) were used for trend analysis.

We used camera trap based mark-recapture framework^15^ to estimate spatially explicit densities of tigers and leopards^25^. We divided our survey area in two regions i.e. Banjar catchment and Halon catchment based on prey densities and logistical reasons. In the initial 2 years 2011 to 2012 our sampling areas were smaller, an area of 280 km^2^ in Banjar catchment and 180 km^2^ in Halon catchment with average trap spacing of 1.8 km (Table 2). Later, from 2013 to 2016, with availability of additional resources, we were able to sample the entire National Park of 916 km^2^ (Fig. 3). We conducted extensive sign surveys to select the best possible location of camera traps. Two camera traps were placed at a single location to photo-capture both flanks of each animal that passed between them. Cameras were placed on forest roads, animal trails and dry streams that were intensively used by tigers and leopards to maximize their detections^15^. We identified individual tigers and leopards from their pelage pattern and prepared capture histories for each individual using Program Extract Compare^45^ and Hotspotter^46^.

We used maximum likelihood based spatially explicit capture recapture (SECR) to estimate densities^25^.The basic parameters for this model are detection probability at the home-range centre, g_0_ and spatial scale of detection, σ. For SECR animals are assumed to be distributed independently in space and occupy home-ranges. The model incorporates spatial locations of captures to estimate detection probability (g) as a declining function of increasing distance (σ) from the animal’s activity centre^47^ akin to distance sampling^48^. We analysed our data using package “secr” (version 3.1.8)^38^ on R platform. For estimating trends in density, we used data from the area that was consistently sampled across all years. Capture histories for tigers and leopards were recorded for both blocks separately between 2011 to 2016 as well as for the entire Kanha National Park between 2013 to 2016. We used multisession model of SECR to estimate densities and their trends across all sessions (years), to compute the finite rate of increase (λ) by fitting session as a predictor in the model^38^. We used an 8 km buffer around the outermost trap locations as model space. The buffer width was decided based on our data, using “suggest.buffer” argument of secr 3.1.8^38^. The suggested buffer was between 7–8 km for both species, hence we choose 8 km buffer width for both tigers and leopards. We removed non-habitat (human settlements) from the buffer to get the final region of model integration, i.e. the habitat mask for estimating density.

Males and females of large felids have different home-range sizes^49^. Hence, sex specific movement could potentially be a source of variability in capture probability^41^. We identified genders of tigers and leopards based on genitalia and secondary sexual characters (nipples) from our long-term dataset. We accounted for potential sources of variability in our data by modelling g_0_ and σ as a function of gender and sampling year (as density was likely to change between years and potentially alter σ).

We used hybrid mixture models of SECR (“hcov” argument in secr) using full likelihood approach to accommodate the unsexed individuals and allocate them to gender classes based on their detection (g_0_) and movement (σ) parameters. The mixing parameter *pmix* indicates and models the detection parameters to the two sexes (male & female) as a two-class mixture. The parameter *pmix* gives us the detection corrected sex ratio as a mixing proportion of the sexes. We selected the best model based on Akaike information criteria corrected for small samples (AICc)^50^.

Movement parameter σ, is often used as a surrogate for home range size^34^. At landscape scale, home-range as indexed by σ, was shown to be density dependent as it declined exponentially with tiger densities^34^. We test this premise at the PA scale^22^ by modelling σ as a declining function of tiger and leopard density. For regression analysis a prerequisite is that x values (independent variables) are known with certainty^51^. In our case, the independent variable i.e. tiger and leopard density were estimates, where leopard densities in particular had large variances (see results) making inference from simple regression analysis questionable. To address this discrepancy, and ascertain that the pattern we observed from our regression model (σ versus density) was not due to chance variation in our data, we simulated 100 values of σ and density for both sexes of tigers and leopards for each year using the mean and standard deviation of our estimates. We ran 100 regression models by randomly choosing from this simulated data of σ and density and computed the average slope, R^2^ values, and their 95% confidence intervals. If our hypothesis of declining sigma with increasing density were true, then the 95% confidence intervals on the slope of the regressions would be negative.

Since SECR models are spatially explicit in nature, they produce fine scale density maps^52^. We generated spatial variation maps of tiger and leopard densities within the sampled area and a buffer of 3 km width (equal to one σ of tiger and leopard). We also estimated population size of both tigers and leopards within this same region^53^.

To understand how leopard population responds to the density and growth of tigers at the scale of a home-range (10 km^2^) of breeding tigresses^54^, we extracted the density and computed growth rates (*r*) of tigers and leopards in each grid of 10 km^2^ from data between 2013 to 2016 for entire Kanha National Park. For each grid, we estimated the growth rate (*r*) for both carnivores by regressing Natural log of density against years^55^. We plotted leopard density and growth rates (heat and contour plots) against tiger density and tiger growth rates to evaluate the demographic response of leopards to tiger demography.

## Supplementary information

Supplementary Material

## Data Availability

Photo-capture matrices of tigers and leopards are not publicly available due to threat of poaching. All other data are either in the main paper or in the Supplementary Material.
