# Supplementary material for: Do conservation strategies that increase tiger populations have consequences for other wild carnivores like leopards?
Source: Sci Rep. 2019 Oct 11;9:14673. doi: 10.1038/s41598-019-51213-w (PMC6789119; doi:10.1038/s41598-019-51213-w)

**Supplementary Material**

**Do conservation strategies that increase tiger populations have consequences for other wild carnivores like leopards?**

**Ujjwal Kumar**^1,^**, Neha Awasthi**^1^**, Qamar Qureshi**^1^ **and Yadvendradev Jhala**^1^

^1^Wildlife Institute of India, Tiger Cell, Chandrabani, Dehradun, 248001, India.

| Site | Year | **Tiger** | | | | **Leopard** | | | |
| --- | --- | --- | --- | --- | --- | --- | --- | --- | --- |
|  |  | Mean Maximum Distance Moved (± SE) Km | Maximum Distance Moved (Km) | Detections at same locations  (No.) | Detection at different locations  (No.) | Mean Maximum Distance Moved (± SE) Km | Maximum Distance Moved (Km) | Detections at same locations  (No.) | Detection at different location  (No.) |
| Banjar Catchment | 2011 | 5.46 ± 0.69 | 15.45 | 57 | 223 | 4.76 ± 1.19 | 19.8 | 14 | 50 |
|  | 2012 | 4.9 ± 0.64 | 11.35 | 55 | 165 | 4.7 ± 1.7 | 18.9 | 12 | 53 |
|  | 2013 | 4.8 ± 0.42 | 10.47 | 49 | 201 | 3.7 ± 0.9 | 10.0 | 18 | 58 |
|  | 2014 | 5.7 ± 0.39 | 10.52 | 90 | 822 | 3.0 ± 0.43 | 5.9 | 17 | 83 |
|  | 2015 | 5.0 ± 0.44 | 12.6 | 118 | 523 | 3.8 ± 0.66 | 7.3 | 9 | 36 |
|  | 2016 | 4.7 ± 0.34 | 10.28 | 90 | 712 | 4.0 ± 0.53 | 8.1 | 40 | 180 |
|  | | | | | | | | | |
| Halon Catchment | 2011 | 7.8 ± 1.12 | 11.29 | 6 | 106 | 3.1 ± 0.7 | 8.2 | 14 | 32 |
|  | 2012 | 7.4 ± 0.93 | 13.29 | 12 | 168 | 3.4 ± 1.02 | 12.0 | 11 | 32 |
|  | 2013 | 6.8 ± 1.29 | 8.91 | 8 | 103 | 2.9 ± 0.81 | 8.0 | 11 | 45 |
|  | 2014 | 8.8 ± 0.73 | 9.21 | 58 | 204 | 4.0 ± 0.76 | 8.5 | 5 | 67 |
|  | 2015 | 9.0 ± 1.22 | 10.89 | 14 | 139 | 3.6 ± 0.52 | 6.6 | 9 | 81 |
|  | 2016 | 13.3 ± 5.3 | 10.93 | 8 | 189 | 3.89 ± 0.5 | 10.8 | 49 | 192 |
|  | | | | | | | | | |
| Kanha National Park | 2013 | 6.3 ± 0.4 | 13.8 | 58 | 424 | 5.7 ± 0.56 | 10.4 | 46 | 147 |
|  | 2014 | 6.7 ± 0.39 | 15 | 179 | 1331 | 4.2 ± 0.42 | 21.0 | 49 | 387 |
|  | 2015 | 7.2 ± 0.65 | 25.19 | 174 | 943 | 5.5 ± 0.84 | 28.6 | 28 | 222 |
|  | 2016 | 6.69 ± 0.46 | 14.29 | 128 | 1287 | 5.26 ± 0.49 | 22.4 | 119 | 711 |

Table S1. Movement distances and detections of tiger and leopard in Kanha National Park and catchments between 2011-2016

| **Site** | **Species** | **model** | **npar** | **logLik** | **AIC** | **AICc** | **dAICc** | **AICcwt** |
| --- | --- | --- | --- | --- | --- | --- | --- | --- |
| Kanha National Park | Tiger | D~Session g0~h_2_ σ~session + h_2_ pmix~h_2_ | 10 | -10394.80 | 20809.60 | 20810.49 | 0.00 | 0.94 |
|  |  | D~Session g0~h_2_ σ~session + h2 pmix~session + h_2_ | 13 | -10394.19 | 20814.38 | 20815.87 | 5.38 | 0.06 |
|  |  | D~Session g0~1 σ~session + h_2_ pmix~session + h_2_ | 12 | -10421.76 | 20867.52 | 20868.79 | 58.30 | 0.00 |
|  |  | D~Session g0~h_2_ σ~h_2_ pmix~h_2_ | 7 | -10483.09 | 20980.17 | 20980.62 | 170.13 | 0.00 |
|  |  | D~Session g0~1 σ~h_2_ pmix~h_2_ | 6 | -10511.90 | 21035.81 | 21036.14 | 225.65 | 0.00 |
|  |  | D~Session g0~1 σ~1 pmix~h_2_ | 5 | -10828.44 | 21666.88 | 21667.12 | 856.63 | 0.00 |
|  |  | | | | | | | |
|  | Leopard | D~Session g0~h_2_ σ~session + h_2_ pmix~h_2_ | 10 | -5697.74 | 11415.49 | 11416.17 | 0.00 | 0.88 |
|  |  | D~Session g0~h_2_ σ~session + h_2_ pmix~session + h_2_ | 13 | -5696.51 | 11419.02 | 11420.17 | 4.00 | 0.12 |
|  |  | D~Session g0~1 σ~session + h_2_ pmix~session + h_2_ | 12 | -5704.59 | 11433.18 | 11434.17 | 17.99 | 0.00 |
|  |  | D~Session g0~h_2_ σ~h_2_ pmix~h_2_ | 7 | -5721.64 | 11457.28 | 11457.63 | 41.45 | 0.00 |
|  |  | D~Session g0~1 σ~h_2_ pmix~h_2_ | 6 | -5733.80 | 11479.60 | 11479.86 | 63.69 | 0.00 |
|  |  | D~Session g0~1 σ~1 pmix~h_2_ | 5 | -6058.24 | 12126.48 | 12126.67 | 710.50 | 0.00 |

Table S2 . Competing models and model selection statistics for SECR analysis of tiger and leopard photo-capture histories in Kanha National Park.

*npar = No. of parameters, Session= sampling session (year) used as predictor,* g_0_ = Detection probability at home range centre, σ = Movement parameter sigma, session *=* variable for each sampling year*, pmix =* Mixing proportion of two sexes, *h_2_ = Sex*

Table S3. Competing models and model selection statistics for SECR analysis of tiger and leopard photo-capture histories in Banjar & Halon catchments of Kanha Tiger Reserve.

| **Site** | **Species** | **model** | **npar** | **logLik** | **AIC** | **AICc** | **dAICc** | **AICcwt** |
| --- | --- | --- | --- | --- | --- | --- | --- | --- |
| Banjar Catchment | Tiger | D~Session g0~ h_2_ σ~session + h_2_ *pmix*~h_2_ | 12 | -6183.79 | 12391.58 | 12392.89 | 0.00 | 1.00 |
|  |  | D~Session g0~h_2_ σ~session + h_2_ *pmix*~session + h_2_ | 17 | -6184.28 | 12402.56 | 12405.18 | 12.29 | 0.00 |
|  |  | D~Session g0~1 σ~session + h_2_ *pmix*~session + h_2_ | 16 | -6219.18 | 12470.36 | 12472.67 | 79.78 | 0.00 |
|  |  | D~Session g0~h2 σ~h_2_ *pmix*~h_2_ | 7 | -6262.95 | 12539.89 | 12540.35 | 147.46 | 0.00 |
|  |  | D~Session g0~1 σ~h_2_ *pmix*~h_2_ | 6 | -6315.21 | 12642.42 | 12642.76 | 249.87 | 0.00 |
|  |  | D~Session g0~session σ~session *pmix*~h_2_ | 15 | -6376.47 | 12782.95 | 12784.98 | 392.09 | 0.00 |
|  |  | D~Session g0~1 σ~1 *pmix*~h_2_ | 5 | -6572.04 | 13154.08 | 13154.32 | 761.43 | 0.00 |
|  |  |  |  |  |  |  |  |  |
|  | Leopard | D~Session g0~1 σ~session + h_2_ *pmix*~session + h_2_ | 16 | -1899.85 | 3831.70 | 3835.47 | 0.00 | 0.64 |
|  |  | D~Session g0~h_2_ σ~session + h_2_ *pmix*~session + h_2_ | 17 | -1899.84 | 3833.69 | 3837.96 | 2.49 | 0.18 |
|  |  | D~Session g0~h2 σ~session + h_2_ *pmix*~h_2_ | 12 | -1905.95 | 3835.91 | 3838.01 | 2.54 | 0.18 |
|  |  | D~Session g0~1 σ~h_2_ *pmix*~h_2_ | 6 | -1988.38 | 3988.76 | 3989.30 | 153.83 | 0.00 |
|  |  | D~Session g0~h_2_ σ~h_2_ *pmix*~h_2_ | 7 | -1987.98 | 3989.96 | 3990.69 | 155.22 | 0.00 |
|  |  | D~Session g0~session σ~session *pmix*~h_2_ | 15 | -2039.31 | 4108.62 | 4111.93 | 276.46 | 0.00 |
|  |  | D~Session g0~1 σ~1 *pmix*~h_2_ | 5 | -2150.93 | 4311.87 | 4312.26 | 476.78 | 0.00 |
|  | | | | | | | | |
| Halon Catchment | Tiger | D~Session g0~h_2_ σ~session + h_2_ *pmix*~h_2_ | 12 | -2144.59 | 4313.18 | 4318.95 | 0.00 | 0.84 |
|  |  | D~Session g0~h_2_ σ~h_2_ *pmix*~h_2_ | 7 | -2153.23 | 4320.47 | 4322.37 | 3.41 | 0.15 |
|  |  | D~Session g0~1 σ~h_2_ *pmix*~h_2_ | 6 | -2157.26 | 4326.52 | 4327.92 | 8.96 | 0.01 |
|  |  | D~Session g0~h_2_ σ~session + h_2_ *pmix*~session + h_2_ | 17 | -2141.64 | 4317.28 | 4329.77 | 10.82 | 0.00 |
|  |  | D~Session g0~1 σ~session + h_2_ *pmix*~session + h_2_ | 16 | -2145.78 | 4323.57 | 4334.45 | 15.49 | 0.00 |
|  |  | D~Session g0~session σ~session *pmix*~h_2_ | 15 | -2168.98 | 4367.96 | 4377.37 | 58.42 | 0.00 |
|  |  | D~Session g0~1 σ~1 *pmix*~h_2_ | 5 | -2184.53 | 4379.05 | 4380.04 | 61.08 | 0.00 |
|  |  |  |  |  |  |  |  |  |
|  | Leopard | D~Session g0~h_2_ σ~session + h2 *pmix*~h_2_ | 12 | -1570.91 | 3165.82 | 3168.82 | 0.00 | 0.87 |
|  |  | D~Session g0~1 σ~h_2_ *pmix*~h_2_ | 6 | -1580.27 | 3172.54 | 3173.31 | 4.48 | 0.09 |
|  |  | D~Session g0~h_2_ σ~h_2_ *pmix*~h_2_ | 7 | -1580.09 | 3174.19 | 3175.21 | 6.39 | 0.04 |
|  |  | D~Session g0~1 σ~session + h_2_ *pmix*~session + h_2_ | 16 | -1570.73 | 3173.45 | 3178.89 | 10.07 | 0.00 |
|  |  | D~Session g0~h_2_ σ~session + h_2_ *pmix*~session + h_2_ | 17 | -1570.14 | 3174.28 | 3180.46 | 11.64 | 0.00 |
|  |  | D~Session g0~session σ~session *pmix*~h_2_ | 15 | -1642.52 | 3315.04 | 3319.80 | 150.97 | 0.00 |
|  |  | D~Session g0~1 σ~1 *pmix*~h_2_ | 5 | -1659.81 | 3329.62 | 3330.16 | 161.34 | 0.00 |

*npar = No. of parameters, Session= sampling session used as predictor,* g_0_ = Detection probability at home range centre, σ = sigma Movement parameter, session *=* variable for each sampling year*, pmix =* Mixing proportion of two sexes, *h_2_ =* Sex as mixture

Table S4. Results of 100 simulated regression analysis from a random sample of density and sigma picked for each year from the distribution of density and sigma for male and female tigers and leopards. Confidence Intervals (95% CI) of all regression slopes did not include zero, suggesting that sigma declined with increasing density for both genders of tigers and leopards

| **Species & Gender** | **parameter** | **mean** | **95% lower CI** | **95% upper CI** |
| --- | --- | --- | --- | --- |
| Male Tiger | slope | -0.15 | -0.15 | -0.14 |
|  | R^2^ | 0.74 | 0.73 | 0.76 |
| Female Tiger | slope | -0.14 | -0.15 | -0.13 |
|  | R^2^ | 0.63 | 0.59 | 0.67 |
| Male Leopard | slope | -0.18 | -0.19 | -0.17 |
|  | R^2^ | 0.38 | 0.35 | 0.41 |
| Female Leopard | slope | -0.10 | -0.11 | -0.09 |
|  | R^2^ | 0.37 | 0.34 | 0.39 |


Supplement: Supplementary file 1 — Supplementary Material [file 41598_2019_51213_MOESM1_ESM.docx]
